# Scoring Model to Predict Functional Outcome in Poor-Grade Aneurysmal Subarachnoid Hemorrhage

**DOI:** 10.3389/fneur.2021.601996

**Published:** 2021-02-18

**Authors:** Jie Shen, Jianbo Yu, Sicong Huang, Rajneesh Mungur, Kaiyuan Huang, Xinfa Pan, Guofeng Yu, Zhikai Xie, Lihui Zhou, Zongchi Liu, Dexin Cheng, Jianwei Pan, Renya Zhan

**Affiliations:** ^1^Department of Neurosurgery, College of Medicine, The First Affiliated Hospital, Zhejiang University, Hangzhou, China; ^2^Department of Neurosurgery, Quzhou People's Hospital, Quzhou, China

**Keywords:** scoring system, prognosis, poor-grade, aneurysmal subarachnoid hemorrhage, model

## Abstract

**Background:** Patients with poor-grade aneurysmal subarachnoid hemorrhage (aSAH), defined as World Federation of Neurosurgical Societies (WFNS) grades IV–V have high rates of disability and mortality. The objective of this study was to accurately prognosticate the outcomes of patients with poor-grade aSAH by developing a new scoring model.

**Methods:** A total of 147 poor-grade aSAH patients in our center were enrolled. Risk variables identified by multivariate logistic regression analysis were used to devise a scoring model (total score, 0–9 points). The scores were estimated on the basis of β coefficients. A cohort of 68 patients from another institute was used to validate the model.

**Results:** Multivariate logistic regression analysis revealed that modified Fisher grade >2 [odds ratio [OR], 2.972; *P* = 0.034], age ≥65 years (OR, 3.534; *P* = 0.006), conservative treatment (OR, 5.078; *P* = 0.019), WFNS grade V (OR, 2.638; *P* = 0.029), delayed cerebral ischemia (OR, 3.170; *P* = 0.016), shunt-dependent hydrocephalus (OR, 3.202; *P* = 0.032), and cerebral herniation (OR, 7.337; *P* < 0.001) were significant predictors for poor prognosis [modified Rankin Scale [mRS] ≥3]. A scoring system was constructed by the integration of these factors and divided the poor-grade aSAH patients into three categories: low risk (0–1 points), intermediate risk (2–3 points), and high risk (4–9 points), with predicted risks of poor prognosis of 11, 52, and 87%, respectively (*P* < 0.001). The area under the curve in the derivation cohort was 0.844 (95% CI, 0.778–0.909). The AUC in the validation cohort was 0.831 (95% CI, 0.732–0.929).

**Conclusions:** The new scoring model can improve prognostication and help decision-making for subsequent complementary treatment in patients with aSAH.

## Introduction

Intracranial aneurysms are abnormal protrusions of the intracranial arterial wall arising from various causes (1, 2). The prevalence rate of intracranial aneurysms in the global population (mean age, 50 years) is up to 3.2% (3). A previous report described that approximately, 1–2% of these aneurysms will rupture (4). According to statistics, the global incidence of aneurysmal subarachnoid hemorrhage (aSAH) is 9–11 per 100,000 people/year. Furthermore, poor-grade aSAH [World Federation of Neurological Surgeons [WFNS] grades IV–V] accounts for 18–30% of all aSAH cases (5, 6). A meta-analysis by Han et al. (7) reported a 26% mortality rate for poor-grade aSAH. At present, most related literature indicates that the disability rate for poor-grade aSAH exceeds 60% (8).

Regarding prognosis prediction, the International Subarachnoid Aneurysm Trial (ISAT) could achieve an accurate prediction of 60-day mortality after aSAH (9). Meanwhile, the Subarachnoid Hemorrhage International Trialists (SAHIT) model successfully predicted long-term outcomes and was used to counsel patients with aSAH and their family members (10). An external validation of the SAHIT model using the Barrow Ruptured Aneurysm Trial (BRAT) cohort revealed that its area under the curve (AUC) for unfavorable outcomes was 0.734 (11). It is worth pointing out that these studies included patients exposed to different subgroups of various treatment procedures, and that most of them were good-grade aSAH patients eligible for surgical treatment. Although good-grade and poor-grade aSAH patients differ in disease progression and survival prognosis (5, 12), previous studies typically combined these patients for analysis without detailed stratification (9–11). Therefore, the previous predictive models have some limitations for the accurate prediction of outcomes in poor-grade aSAH patients. The objective of the present study was to devise a new scoring system that can evaluate the prognosis of patients with poor-grade aSAH intuitively.

## Materials and Methods

### Study Design

The derivation cohort comprised poor-grade aSAH patients who were treated in the Department of Neurosurgery at our center from January 2013 to January 2019. The validation cohort was composed of aSAH patients treated in the Department of Neurosurgery at another institute from January 2016 to January 2019. The inclusion criteria were: (1) aSAH diagnosed by computed tomography (CT) or lumbar puncture in the medical center; (2) aneurysm confirmed as the cause of SAH on digital subtraction angiography (DSA), three-dimensional CT angiography, or magnetic resonance angiography; (3) WFNS grade IV and V; (4) signed informed consent from family members of patients to cooperate with clinical treatment procedures; and (5) patients without surgical treatment in referral centers. The exclusion criteria were: (1) traumatic, mycotic, or arteriovenous malformation-related aneurysms or SAH of unknown etiology; (2) WFNS grade less than or equal to III; (3) absence of important medical information for patients; and (4) patients treated with medical instruments or drugs that were not approved. The STROBE statement guideline has been implemented in this manuscript.

### Clinical Therapeutic Protocol

All patients admitted under emergency conditions received early resuscitation, early CT angiography, multidisciplinary consensus consultation, conservative treatment, or surgical treatment. A multidisciplinary team of neurosurgeons and anesthesiologists made therapeutic decisions on the basis of the clinical conditions and family members' consent. The treatment mode in our study was divided into two categories: (1) the conservative group: patients who received pure medicinal conservative treatment or patients who received other basic surgical methods without treatment of the underlying aneurysm, such as external drainage surgery, hematoma evacuation, and decompressive craniectomy; and (2) the clipping or coiling group: patients who underwent primary aneurysm embolization or clipping alone, or combined with a basic surgical operation involving coiling or clipping. Patients underwent surgical treatment in accordance with an early treatment strategy (within 72 h). All aSAH patients were treated with routine SAH treatments, including mannitol, anticonvulsants, triple-H (hypervolemia/hypertension/hemodilution) treatment, and nimodipine treatment. Antiplatelets were administered to prevent thrombosis after stent-assisted embolization.

### Clinical Data and Variable Definitions

The clinical variables were collected retrospectively from the hospital database. Patient's baseline information and imaging information were collected by two doctors separately, and any conflicting items were evaluated again by a senior doctor. Age was divided into two subcategories in accordance with the cutoff age of 65 years. The modified Fisher grade was divided into two subcategories in accordance with the cutoff value of grade 2. A wide-necked aneurysm was defined as an aneurysm with a neck width ≥4 mm or a neck ratio exceeding 1:2. Cerebral herniation was diagnosed based on CT results and corresponding signs, including deterioration of consciousness disturbance, some focal signs, oculomotor palsy, respiratory distress, and decorticate or decerebrate rigidity (13). Among the complications, shunt-dependent hydrocephalus (SDH) was defined as clinical deterioration occurring on the 14th day after aSAH and no other causes were found except for hydrocephalus, at the same time, it was observed in CT that the ventricular size progressively increased and the Evans index exceeded 0.30 (14). Epilepsy was defined as rhythmic jerking, with or without preceding tonic spasms, that was focal or generalized in nature, with or without loss of consciousness. Even one late seizure was considered to be post-stroke epilepsy (15, 16). Aneurysm rebleeding was defined as a sudden clinical deterioration accompanied by increased subarachnoid, intracerebral, or ventricular blood flow on subsequent CT scans (17). Cerebral vasospasm (CVS) was defined as arterial stenosis found on the CT angiography examination when the patient's clinical symptoms deteriorated, or vasospasm was detected during DSA (18). Delayed cerebral ischemia (DCI) was defined as: (1) occurrence of focal neurological impairment or decrease of ≥ 2 points on the Glasgow Coma Scale that could not be attributed to another cause, such as cerebral rebleeding or encephaledema; or (2) a new low-density area not seen on the previous CT scan and not attributable to other causes such as surgical treatment, or a low-density shadow after absorption of a hematoma (19).

### Outcome Measures

A dynamic follow-up evaluation was performed at 6 months after discharge by neurosurgeons in accordance with the modified Rankin score (mRS) via telephone call or outpatient appointment. The assessment of neurological prognosis mainly focused on whether or not the patients presented with self-care ability. Functional prognosis was classified as good (mRS scores 0–2) or poor (mRS scores 3–6).

### Statistical Analysis

Data were analyzed using the SPSS Version 23.0 software (IBM, Armonk, NY). Continuous variables were reported as mean ± standard deviation and compared between favorable and poor outcomes using an unpaired *t*-test. Categorical variables were reported as proportion and percentile and analyzed by the chi-square or Fisher exact test, as appropriate. Univariate and multivariate logistic regression analyses were performed using poor outcomes as the outcome variable in the derivation cohort. Variables with *P* ≤ 0.1 in the univariate analyses were entered into the multivariate logistic regression analysis with stepwise backward selection. Risk variables independently associated with prognosis were entered into the new scoring model. The points for individual factors were assigned on the basis of their corresponding β coefficients in the multivariate analysis. The discrimination of the prognostic model was assessed by the AUC in a receiver operating characteristic curve analysis. The Hosmer–Lemeshow goodness-of-fit test and a calibration plot were used to evaluate the calibration of the prediction model.

## Results

### Basic Information of Patients

**Figure 1:**
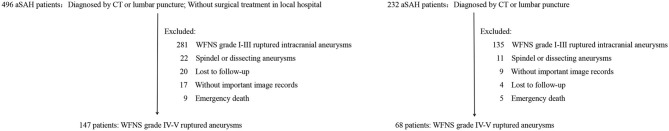
Study flow diagram.

The detailed processes for the selection and exclusion of patients in the derivation group and validation group are shown in Figure 1. In total, 147 patients were included in the derivation study and 68 patients were included in the validation cohort.

**Figure 2:**
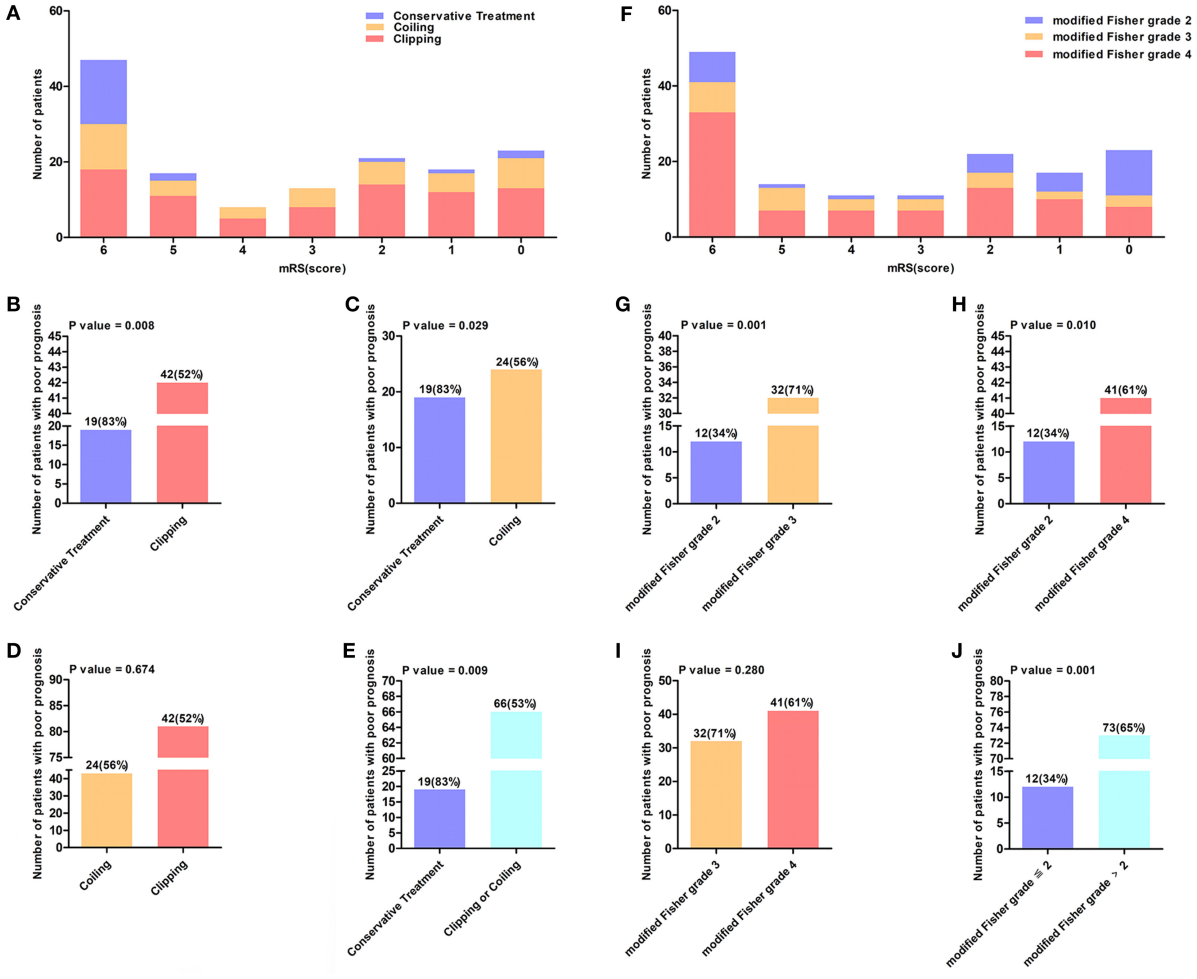
**(A)** The distribution of mRS score of 147 poor-grade aSAH patients who accepted different treatment methods. The value above the histogram shows the number of patients with poor prognosis and their percentage, for instance, the interpretation of 19 (83%) in **(B)** is that 19 (83%) patients had a poor outcome among 23 patients who received conservation treatment. **(B–E)** Reflects the influence of different treatment methods on the prognosis of patients. **(F)** Shows the distribution of mRS score of 147 poor-grade aSAH patients in different modified Fisher grade. **(G–J)** Reflects the influence of different modified Fisher grade groups on the prognosis of patients.

In the derivation cohort, 55 (37%) patients were male and 92 (63%) were female. Among these patients, the age range was 37–87 years, the mean age was 61.3 ± 11.5 years, and ~39% were aged ≥65 years. The baseline characteristics of the 147 patients with poor-grade aSAH are presented in Table 1. In total, 124 (84.3%) patients received surgical therapies including coiling (29.2%) and clipping (55.1%), and 23 (15.7%) patients received conservative treatment. In addition, there was no significant statistical difference between the treatment approach and the WFNS grade (*P* = 0.110). The distribution of mRS scores among the 147 poor-grade aSAH patients with different treatments is shown in Figure 2A. As shown in Figures 2B–E, patients who received coiling or clipping had a better prognosis than patients who received conservative treatment, but there was no significant difference in prognosis between patients who received coiling or clipping. There were 114 (77.6%) poor-grade aSAH patients with a modified Fisher grade >2 and 85 (57.8%) patients with WFNS grade V. The distribution of mRS scores among the 147 poor-grade aSAH patients with different modified Fisher grades is shown in Figure 2F. The influences of different modified Fisher grades on the prognosis of patients are shown in Figures 2G–J. During the 6-month follow-up after discharge, 85 patients (58%) had poor outcomes.

**Table 1:** Demographic and baseline characteristics of the study population and univariate analysis results of modeling cohorts.

**Variable**	**Derivation cohort**		***P-*value (Modeling cohort)**	**Validation cohort**	
	**Favorable outcome**	**Poor outcome**		**Favorable outcome**	**Poor outcome**
	**%/Mean ± SD**	**%/Mean ± SD**		**%/Mean ± SD**	**%/Mean ± SD**
**No. of patients**	**62 (42%)**	**85 (58%)**		**30 (44%)**	**38 (56%)**
**Demographic characters**					
**Age (years)**			**0.027**		
<65	44 (71%)	45 (53%)		22 (73%)	21 (55%)
≧65	18 (29%)	40 (47%)		8 (27%)	17 (45%)
**Gender**			0.147		
Male	19 (31%)	36 (42%)		10 (33%)	16 (42%)
Female	43 (69%)	49 (58%)		20 (67%)	22 (58%)
**Medical history**					
**Hyperlipidemia**	19 (31%)	26 (31%)	0.994	7 (23%)	11 (29%)
**Hypertension**	32 (52%)	54 (64%)	0.148	18 (60%)	22 (58%)
**Diabetes mellitus**	14 (23%)	28 (33%)	0.170	8 (27%)	14 (37%)
**Cerebrovascular disease**	8 (13%)	11 (13%)	0.995	5 (17%)	8 (21%)
**Alcohol consumption**	13 (21%)	28 (33%)	0.110	8 (27%)	10 (26%)
**Smoking**	15 (24%)	21 (25%)	0.943	10 (33%)	9 (24%)
**Radiologic imaging and laboratory examination**					
**WBC ≧ 15 × 10**^**9**^	30 (48%)	42 (49%)	0.902	13 (43%)	19 (50%)
**ICH**	21 (34%)	34 (40%)	0.448	10 (33%)	18 (47%)
**IVH**	41 (66%)	73 (86%)	**0.005**	16 (53%)	28 (74%)
**Ventricular casting**	10 (16%)	21 (25%)	0.195	4 (13%)	8 (21%)
**Modified Fisher grade**			**0.001**		
2	23 (37%)	12 (14%)		15 (50%)	9 (24%)
3	13 (21%)	32 (38%)		5 (17%)	11 (29%)
4	26 (42%)	41 (48%)		10 (33%)	18 (47%)
**WFNS**			**<0.001**		
IV	38 (61%)	24 (28%)		15 (50%)	13 (34%)
V	24 (39%)	61 (72%)		15 (50%)	25 (66%)
**Aneurysm morphology**					
**Wide-necked aneurysm**	31 (50%)	49 (58%)	0.317	13 (43%)	19 (50%)
**Multiple aneurysms**	8 (13%)	16 (19%)	0.338	5 (17%)	10 (26%)
**Aneurysm size (mm)**	5.9 ± 3.3	6.1 ± 3.7	0.860	6.5 ± 2.5	6.7 ± 3.1
**Location of aneurysm**			0.435		
ICA	13 (21%)	10 (12%)		6 (20%)	5 (13%)
ACA	6 (10%)	10 (12%)		4 (13%)	3 (8%)
AComA	12 (19%)	21 (25%)		8 (27%)	8 (21%)
MCA	15 (24%)	15 (17%)		5 (17%)	9 (24%)
PComA	11 (18%)	16 (19%)		5 (17%)	8 (21%)
PC	5 (8%)	13 (15%)		2 (6%)	5 (13%)
**Treatment**					
**Therapeutic strategy**			**0.009**		
Coiling	19 (31%)	24 (28%)		16 (53%)	11 (29%)
Clipping	39 (63%)	42 (49%)		9 (30%)	13 (34%)
Conservative treatment	4 (6%)	19 (23%)		5 (17%)	14 (37%)
**CLSD**	21 (34%)	28 (33%)	0.906	7 (23%)	9 (24%)
**Complication**					
**Acute hydrocephalus**	12 (19%)	20 (24%)	0.545	7 (23%)	10 (26%)
**SDH**	8 (13%)	22 (26%)	**0.054**	7 (23%)	12 (32%)
**Aneurysm rebleeding**	1 (2%)	13 (15%)	**0.004**	0	8 (21%)
**Epilepsy**	4 (7%)	9 (11%)	0.383	1 (3.3%)	3 (8%)
**Pulmonary infection**	32 (52%)	50 (59%)	0.385	16 (53%)	21 (55%)
**Intracranial infection**	33 (53%)	40 (47%)	0.460	13 (43%)	16 (42%)
**CVS**	7 (11%)	21 (25%)	**0.041**	7 (23%)	16 (42%)
**DCI**	13 (21%)	32 (38%)	**0.030**	6 (20%)	11 (29%)
**Cerebral herniation**	6 (10%)	38 (45%)	**<0.001**	2 (7%)	16 (42%)

*ICH, intracerebral hemorrhage; IVH, intraventricular hemorrhage; WFNS, world federation of neurosurgical societies; CLSD, continuous lumbar subarachnoid drainage; SDH, shunt-dependent hydrocephalus; CVS, cerebral vasospasm; DCI, delayed cerebral ischemia; ICA, internal carotid artery; ACA, anterior cerebral artery; AComA, Anterior communicating artery; MCA, middle cerebral artery; PComA, posterior communicating artery; PC, posterior cerebral circulation. P values less than 0.1 are shown in bold*.

Of the 68 patients in the validation cohort, 25 (36.7%) patients were aged ≥65 years. A total of 44 (64.7%) poor-grade aSAH patients had a modified Fisher grade >2 and 40 (58.8%) patients presented with WFNS grade V. Forty-nine (72%) patients underwent surgical therapies. At the 6-month follow-up after discharge, 38 (56%) patients had poor outcomes. Specific data for the validation cohort are presented in Table 1.

### Univariate Analyses of Poor Outcomes

The associations between clinical variables and poor outcomes identified by univariate analyses are shown in Table 1. Poor prognosis was associated with age ≥65 years (*P* = 0.027), intraventricular hemorrhage (IVH) (*P* = 0.005), WFNS grade V (*P* < 0.001), conservative treatment (*P* = 0.009), modified Fisher grade >2 (*P* = 0.001), emergence of cerebral herniation (*P* < 0.001), aneurysm rebleeding (*P* = 0.004), CVS (*P* = 0.041), and DCI (*P* = 0.030). Medical histories of patients and data for aneurysms were not significantly correlated with clinical outcomes.

### Multivariate Regression Analysis of Poor Outcome

Ten variables with *P* < 0.1 in the univariate analyses were entered into the multivariate logistic regression analysis (Table 2). The results showed that age ≥65 years (OR, 3.534; *P* = 0.006), modified Fisher grade >2 (OR, 2,972; *P* = 0.034), cerebral herniation (OR, 7.337; *P* < 0.001), WFNS grade V (OR, 2.638; *P* = 0.029), SDH (OR, 3.202; *P* = 0.032), conservative treatment (OR, 5.078; *P* = 0.019), and DCI (OR, 3.170; *P* = 0.016) were independent risk factors for poor outcomes. The Hosmer–Lemeshow test reflected a satisfactory degree of consistency between the predicted risk of the model and the actual risk (*P* = 0.589; Table 2).

**Table 2:** Multivariate logistic regression model for poor prognosis risk of poor-grade aSAH.

**Variable included in model**	**S.E**	**OR**	**95%CI**	***P***
Modified Fisher grade (grade 3, 4)	0.515	2,972	1.083–8.156	0.034
Age (≥ 65)	0.457	3.534	1.442–8.662	0.006
Therapeutic strategy (conservation)	0.694	5.078	1.303–19.790	0.019
WFNS (grade V)	0.444	2.638	1.104–6.300	0.029
DCI	0.478	3.170	1.242–8.090	0.016
SDH	0.542	3.202	1.107–9.263	0.032
Cerebral herniation	0.565	7.337	2.426–22.192	<0.001
Hosmer and Lemeshow test				
X^2^				6.525
Degree of freedom				8
*P*				0.589

### Development of the Scoring System

By integration of the seven independent risk factors, namely modified Fisher grade >2, age ≥65 years, conservative treatment, WFNS grade V, DCI, SDH, and cerebral herniation, a scoring system designated Poor-Grade Aneurysmal Subarachnoid Hemorrhage Prognostic Scoring System (PASHPSS) was constructed (Table 3). On the basis of the β coefficients in the multivariate analysis, scores of 2 were assigned to cerebral herniation and conservative treatment, and scores of 1 were assigned to the other five risk factors; otherwise, a score of 0 points was assigned. In accordance with the sum of the scores (range, 0–9), the new model divided poor-grade aSAH patients into three prognostically different categories (Table 4): low risk category, 11% prediction risk of poor prognosis in patients with total scores of 0–1 point; intermediate risk category, 51% prediction risk of poor prognosis in patients with total scores of 2–3 points; high risk category, 87% prediction risk of poor prognosis in patients with total scores of ≥4 points.

**Table 3:** Poor-Grade Aneurysmal Subarachnoid Hemorrhage Prognostic Scoring System (PASHPSS) derived from the β coefficients.

**Variable included in model**	**Categories**	**β coefficient**	**Score**
Modified Fisher grade			
	Below grade 2	0 (reference)	0
	Grade 3, 4	1.09	1
Age			
	<65	0 (reference)	0
	≥ 65	1.26	1
Therapeutic strategy			
	Coiling or clipping	0 (reference)	0
	Conservation	1.63	2
WFNS			
	Grade IV	0 (reference)	0
	Grade V	0.97	1
DCI			
	Non-DCI	0 (reference)	0
	DCI	1.15	1
SDH			
	Non-SDH	0 (reference)	0
	SDH	1.16	1
Cerebral herniation			
	Non-cerebral herniation	0 (reference)	0
	Cerebral herniation	1.99	2

**Table 4:** Risk of poor prognosis for low, intermediate, and high-risk individuals according to the PASHPSS risk score.

**Risk stratification**	**Score**	**Observed risk (validation cohort)**	**Predicted risk**	**OR (95% CI)**
Low risk stratification	0–1	19%	11%	1 (reference)
Moderate risk stratification	2–3	48%	51%	8.6 (2.2–18.7)
High risk stratification	4–9	81%	87%	54.2 (13.2–221.9)

### Discrimination and Calibration of the Scoring System

**Figure 3:**
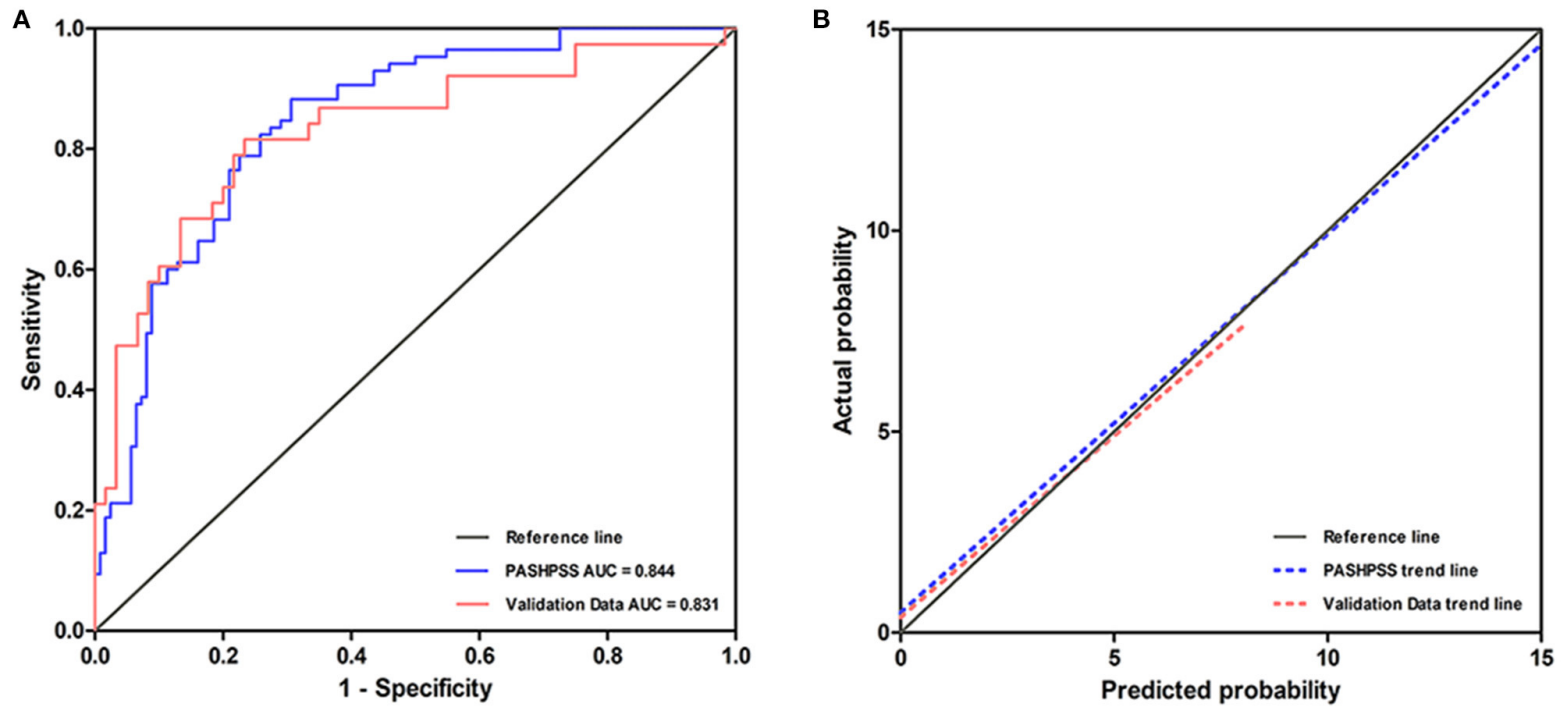
**(A)** The AUC of the PASHPSS is 0.844 (95% CI, 0.778–0.909) in our center's derivation data, while it is 0.831 (95% CI, 0.732–0.929) in validation data. **(B)** A slope of 1 (45 degrees) with an intercept of 0 represents perfect calibration, the deviation from the reference line is smaller, the calibration is better. PASHPSS has a good calibration in derivation cohort and validation cohort.

In the derivation cohort, the AUC of the PASHPSS was 0.844 (95% CI: 0.778–0.909; Figure 3), and the Hosmer–Lemeshow test showed good calibration (*P* = 0.589). In the validation cohort, the PASHPSS also showed good discrimination with an AUC of 0.831 (95% CI, 0.732–0.929; Figure 3) and good calibration by the Hosmer–Lemeshow test (*P* = 0.984). Also in the validation cohort, the observed risks in the three risk categories were close to the predicted risks (Table 3): low risk category, actual observed risk of poor prognosis was 19%; intermediate risk category, actual observed risk of poor prognosis was 48%; high risk category, actual observed risk of poor prognosis was 81%.

## Discussion

As a serious cerebrovascular disease, poor-grade aSAH has high rates of mortality and disability. In this study, the rates of poor prognosis of patients in the modeling cohort and validation cohort both exceeded 55%. Although active and effective treatments can be provided, some aSAH patients still present with neurological dysfunctions and life disorders that have great impacts on society and family members (1, 4, 6, 8). It is necessary to explore the relevant risk factors and evaluate the prognosis of these patients. Several modifiable and non-modifiable risk factors for poor prognosis in poor-grade aSAH patients are currently known, with the most common risk factors being elderly age, cerebral herniation, WFNS grade V, and higher modified Fisher grade (9, 10, 12, 20–24). These risk factors were also identified in the present study.

The choice of treatment method is significantly related to the prognosis of patients with poor-grade aSAH. In a systematic review of 815 patients with aSAH, researchers reported that the rates of good prognosis in patients with clipping, embolization, and conservative treatment were 45.3, 36.3, and 9.0%, respectively (25). In our study, the rate of poor prognosis in patients with clipping or coiling was significantly lower than that in patients with conservative treatment, but there was no statistically significant difference in prognosis between patients with clipping or embolization. Combining our center's experience with previous literature on poor-grade aSAH patients, more aggressive treatment of the underlying aneurysm by surgery is associated with a better therapeutic prognosis than conservative treatment.

Post-operative complications play important roles in the prognosis of poor-grade aSAH patients. As a critical complication, aneurysmal rebleeding usually causes a sharp increase in intracranial pressure, damages the nerve function, and increases the risk of death in the short term (26–28). CVS is generally considered a risk factor for poor prognosis. However, immediate vasospasm is usually difficult to detect, and nimodipine is routinely used in clinical practice to prevent its occurrence, leading to an overall reduction in the incidence of CVS (29). A more commonly observed and easily detected complication during clinical treatment is DCI caused by CVS, which is a strong independent risk factor for poor prognosis in patients with poor-grade aSAH (19, 30, 31). DCI continues to be an important cause of cognitive impairment and disability after aSAH despite aggressive management (32–34). A single-center study on 888 aSAH patients found that SDH was a strong independent risk factor for unfavorable functional outcomes (35). Our final results confirmed predictive roles for the above-mentioned factors.

Some other risk factors have also been raised in recent articles, but have not been widely recognized. IVH was considered as a risk factor for poor outcomes in many reports (36). IVH was identified in our univariate analyses, but subsequently eliminated in the multivariate regression analysis. The possible reason may be that IVH caused impairment of cerebrospinal fluid absorption by blocking arachnoid villi and brain capillaries, thereby affecting the prognosis by developing into chronic hydrocephalus (37, 38). Whether or not aneurysm location and size are predictive factors for poor prognosis of aSAH patients remains inconclusive (24). These inconsistent results may be explained by treatment selection biases in different studies. In the present study, there was no correlation between aneurysm location and size and long-term prognosis. In a multicenter study on poor-grade aSAH patients, Zhao et al. (21) demonstrated that wide-necked aneurysms and post-operative pneumonia were also poor prognostic factors. However, these two risk factors were not identified in our study. Leukocytosis (WBC >15 × 10^9^ /L) was regarded as a predictive factor for poor prognosis in a 9-year cohort study (22), but was not reported in other articles.

Although the current literature on poor-grade aSAH patients has focused on reporting risk factors for prognosis, prognostic predictive models for poor-grade aSAH patients are rare. A recent systematic review assessed 11 clinical prediction models for aSAH patients and found that the most common factors associated with outcomes were age (8 of 11 studies), neurologic grade on admission (10 of 11 studies), and amount of blood detected by CT examination on admission (6 of 11 studies) (24). Although the WFNS and modified Fisher grade scales were commonly used, both scales are not completely reliable in patients because of the subjective nature of the parameters on which the models were built (39). For example, the WFNS and Hunt–Hess scales are generally unreliable in intubated patients. Furthermore, in two articles that established predictive scores in poor-grade aSAH patient populations, the factors were applied, but no additional risk factors were added to circumvent the errors caused by the inter-rater and intra-rater variabilities (22, 23). Undeniably, more valuable risk variables added into a risk score can improve its predictivity. Treatment methods, SDH, and DCI are three factors that affect the long-term neurological prognosis and cognitive impairment of patients, and their roles in predicting the prognosis of patients are worthy of recognition (19, 35, 37, 40). Our PASHPSS showed significantly improved discrimination compared with other risk scores by including these risk factors. For example, the AUC of the SAHIT model was 0.734 (11), while the AUC of the WAP score for poor-grade aSAH patients was 0.74 (23). Meanwhile, the AUC of the PASHPSS was 0.844, which can be regarded as excellent, especially when predicting the prognosis of poor-grade aSAH patients.

At present, several studies have proposed prognosis models for poor-grade aSAH patients, but most of these models have limitations in reporting calibration, discrimination, and external validation. Clinicians generally do not use existing models for the prediction of prognosis in poor-grade aSAH patients, even though their internal effectiveness is not inferior to the PASHPSS (22–24), partly because they lack external validity. However, the PASHPSS showed good discrimination in the validation data. Specifically, its AUC was 0.831, meaning that the system still performed well when it was applied to a new patient cohort.

The present study shows that the PASHPSS developed with identified risk factors can predict the future risk of poor prognosis in aSAH patients very well. Furthermore, it can help guide clinical decisions and patient consultations, and may also reduce the cost of treatment by ensuring effective resource allocation. Such benefits may be particularly important in the management of patients with poor-grade aSAH.

## Limitations

Some limitations of our risk score need to be discussed. First, the statistical data were retrospectively collected. Second, the results of the study only represent the subgroup of poor-grade aSAH patients. Therefore, the scoring model is applicable to the prediction of poor prognosis among poor-grade aSAH patients only. With regard to functional neurologic outcomes, we selected 6 months after discharge as the follow-up point on the basis of the critical period for neurological recovery. However, if data on long-term follow-up can be acquired, the prediction of prognosis will be more accurate. Furthermore, the modeling data were acquired from a single center, which may lead to some inevitable bias in the analysis and conclusions.

## Conclusions

The obtained results have allowed us to draw the following conclusions. The main risk factors affecting the prognosis of patients with poor-grade aSAH are modified Fisher grade, elderly age, therapeutic schedule, WFNS grade, DCI, SDH, and cerebral herniation. The PASHPSS is an efficient tool for predicting the prognosis of poor-grade aSAH, can be easily measured, and is helpful for decision-making on subsequent complementary treatment and in reducing the cost of treatment by ensuring effective resource allocation.

## Data Availability Statement

The raw data supporting the conclusions of this article will be made available by the authors, without undue reservation.

## Ethics Statement

Ethics approval has been obtained from the ethics committee of First Affiliated Hospital of Zhejiang University. Non-essential identifiable details have been omitted from all manuscripts. The patients next of kin provided written informed consent to participate in this study.

## Author Contributions

JS and JY contributed to writing the manuscript, acquisition of the data, and analysis and interpretation of the data. SH contributed to the acquisition of follow-up data and preliminary revision of the manuscript content. RM corrected the English language used in the manuscript. KH contributed to the acquisition of the data and preliminary revision of the manuscript content. XP contributed to preliminary revision of the manuscript content. GY provided the external validation data. ZX, LZ, ZL, and DC contributed to the literature review. JP and RZ contributed to the critical revision of the manuscript for intellectual content. All authors contributed to the article and approved the submitted version.

## Conflict of Interest

The authors declare that the research was conducted in the absence of any commercial or financial relationships that could be construed as a potential conflict of interest.

## Abbreviations

aSAH: Aneurysmal subarachnoid hemorrhage
WFNS: World Federation of Neurosurgical Societies
OR: Odds ratio
CI: Confidence interval
ICU: Intensive care unit
DCI: Delayed cerebral ischemia
CVS: Cerebral vasospasm
GOS: Glasgow Outcome Scale
IVH: Intraventricular hemorrhage
ICH: Intracerebral hemorrhage
CT: Computed tomography
mRS: Modified Rankin score
ISAT: International Subarachnoid Aneurysm Trail
SAHIT: Subarachnoid Hemorrhage International Trialists
BRAT: Barrow Ruptured Aneurysm Trial
CLSD: Continuous lumber subarachnoid drainage
AUC: Area under the curve
WBC: White blood cell count
SDH: Shunt-dependent hydrocephalus
PASHPSS: Poor-Grade Aneurysmal Subarachnoid Hemorrhage Prognostic Scoring System.
